# Surface Finishing of Additive Manufactured Titanium Alloy by Plasma Electrolytic Polishing Without Pretreatments

**DOI:** 10.3390/ma18204719

**Published:** 2025-10-15

**Authors:** Adel Ghezri, Thomas Nelis, Jürgen Burger, Cedric Bessire

**Affiliations:** 1 School of Biomedical and Precision Engineering, Bern University, 3010 Bern, Switzerland; 2 School of Engineering and Computer Science, Bern University of Applied Science, 2501 Biel, Switzerland; 3 Laboratory of Mechanics of Materials and Nanostructures, Empa—Swiss Federal Laboratories for Materials Science and Technology, 3602 Thun, Switzerland

**Keywords:** plasma electrolytic polishing (PEP), additive manufacturing, Ti-6Al-4V alloy

## Abstract

The advent of the additive manufacturing of Ti-6Al-4V (Ti64) alloys has facilitated the production of complex geometries for various industrial applications. Nevertheless, the inherent surface roughness of selective laser melting (SLM)-produced parts remains a critical limitation, adversely affecting fatigue life, wear, corrosion, and compliance with stringent surface quality standards, for example those required in hygienic applications. Conventional post-processing methodologies, encompassing grinding and electropolishing, are frequently multi-stage, labor-intensive, and reliant on hazardous electrolytes, which thus limits their use for certain applications. In this study, plasma electrolytic polishing (PEP) was evaluated as a single-step finishing process for 3D-printed Ti64 components. The findings indicate that PEP efficiently diminished surface roughness from initial values of approximately 9–10 µm to as low as 0.38–0.5 µm within a time frame of 15–20 min, depending on the initial surface condition. These outcomes meet hygienic surface requirements while ensuring the use of environmentally compatible electrolytes. The findings establish PEP as a non-mechanical, efficient, and scalable additive-manufacturing post-processing strategy. It has the capacity to supersede conventional multi-stage workflows and offer substantial reductions in cost, time, and environmental impact.

## 1. Introduction

The advent of additive manufacturing, particularly 3D printing, has precipitated a paradigm shift in the fabrication of complex and customized geometries across diverse industrial sectors [1,2]. Among the materials extensively employed in this domain, Ti-6Al-4V (Ti64) alloy merits particular attention due to its unique mechanical properties, encompassing a high strength-to-weight ratio, exceptional corrosion resistance, and thermal stability [3]. These characteristics make Ti64 a preferred material for applications in the aerospace, automotive, and energy sectors, where performance under demanding conditions is critical [4,5]. The ability of 3D printing to fabricate intricate and tailored designs has unlocked new possibilities for optimizing component performance and reducing material waste. Nevertheless, challenges persist in the post-processing of 3D-printed Ti64 components, particularly in addressing the inherent surface roughness resulting from techniques such as selective laser melting (SLM). SLM-produced surfaces typically exhibit surface roughness values ranging from 9 to 20 µm [6,7], this phenomenon can have a detrimental effect on the functional performance of components, including fatigue resistance [8], wear properties [9], and fluid dynamics [10]. In industries such as pharmaceutical, and medical device manufacturing [11], there is a need to meet stringent surface finish requirements. For instance, the ASME BPE (American Society of Mechanical Engineers: Bioprocessing Equipment) standard [12] stipulates a maximum allowable average surface roughness (Sa) of <0.4 µm for hygienic and high-precision applications [12]. Such standards are also adopted by high-tech industries as benchmarks for low surface roughness. This underscores the necessity for the implementation of effective post-processing methodologies to attain the desired surface quality [13].

Conventional post-processing of Ti64 generally entails a multi-stage workflow, encompassing rough polishing, fine polishing, and a final surface finishing step [14,15]. These methods, such as mechanical polishing, abrasive blasting, grinding, etc., and, for finishing, often electrochemical polishing and chemical etching, are not only labor-intensive and time-consuming but also struggle in these sequential multi-step processes to achieve consistent surface quality, particularly for the complex geometries inherent to 3D-printed parts [16,17]. In contrast, plasma electrolytic polishing (PEP) could replace these multi-step processes as it has shown the potential to reduce surface roughness from SLM initial surfaces to Sa < 0.4 µm while maintaining geometric integrity [17]. Moreover, a considerable proportion of extant electrochemical polishing processes are contingent upon hazardous electrolytes, including hydrofluoric acid (HF), thus giving rise to substantial concerns regarding health, safety, and environmental impact [16,18]. In this context, plasma electrolytic polishing, an advanced surface finishing process, has emerged as a scalable alternative, capable of delivering superior surface refinement and corrosion resistance without the drawbacks of traditional methods [19].

The objective of this study is to show the feasibility of PEP as the sole post-processing method for additively manufactured Ti64 to reach the ASME BPE standard. This approach ensures a substantial reduction in initial surface roughness, achieving values considerably below most established high-tech surface standards. The technique utilizes a less hazardous electrolyte with salts of low concentrations [16], thereby enhancing both safety and environmental sustainability.

## 2. Materials and Methods

For PEP, the anodically polarized workpiece is usually immersed in a cathodic bath containing an aqueous electrolyte [20]. Thereby electrolytes required for PEP are water-based and metal-specific [21], with a typical conductivity of 80 to 140 mS/cm [22]. By applying a DC voltage, a vapor gaseous envelope (VGE) is formed around the workpiece due to joule heating caused by the higher current density on the workpiece. The appearance of the VGE increases the electrical resistance significantly [23] and thus, a plasma is built up inside the VGE around the workpiece. This transient locally instable plasma layer is responsible of polishing the workpiece [24], by both physical plasma and electrochemical processes [14,16,21].

The PEP experiments were conducted under controlled conditions to ensure reproducibility. The electrolyte solution consisted of a mixture of ammonium fluoride (NH_4_F) and ammonium chloride (NH_4_Cl), each at a concentration of maximum 10%, prepared in deionized water. The pH of the electrolyte was meticulously regulated within a range of 5 to 7, a factor which was found to be instrumental in optimizing electrochemical reactions and ensuring system stability. The experiments were conducted under direct current (DC) conditions, with measured current values of around 2 A, which are in accordance with previously reported current densities [25]. The electrolyte temperature value was regulated around 80 °C with a view to enhancing ionic conductivity and reaction kinetics while preventing excessive thermal degradation. A voltage of 300 V was employed to achieve the desired electrochemical polishing effects without inducing unwanted side reactions or system instability. A batch of 20 samples was subjected to testing, with process times of 2, 5, 10, 15, and 20 min, respectively, being systematically evaluated to ascertain the optimal duration for achieving the desired surface finishing.

**Figure 1:**
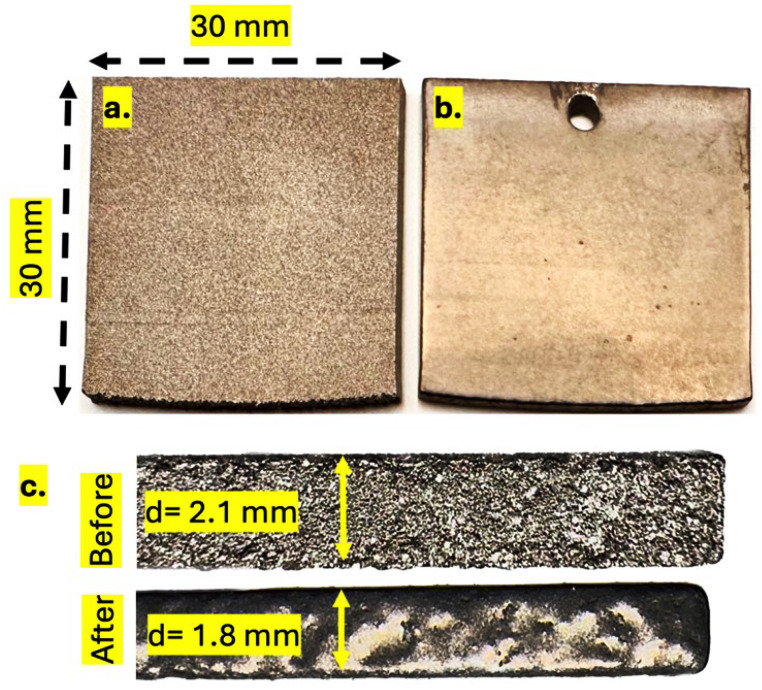
Images of Ti64 3D-printed samples: (**a**) untreated, (**b**) after 20 min of PEP-Bath treatment, and (**c**) the bottom face highlighting the surface etching difference before and after treatment.

The samples utilized in this study (Figure 1a) were fabricated employing selective laser melting (SLM) utilizing conventional process parameters. The laser power was set to 200 W, the scan speed to 1200 mm/s, the hatch spacing to 100 µm, and the layer thickness to 30 µm, corresponding to a volumetric energy density of approximately 70 J/mm^3^ [26]. The process was conducted under an argon atmosphere with an oxygen content of less than 0.2% [27], and the build platform was preheated to 200 °C to reduce residual stresses [26,28]. The experimental conditions resulted in the formation of dense Ti-6Al-4V parts, followed by hot isostatic pressing (HIP) to ensure a dense and homogeneous material structure [29]. Certain samples were subsequently processed for 10 min by ceramic blasting.

## 3. Results and Discussion

**Figure 2:**
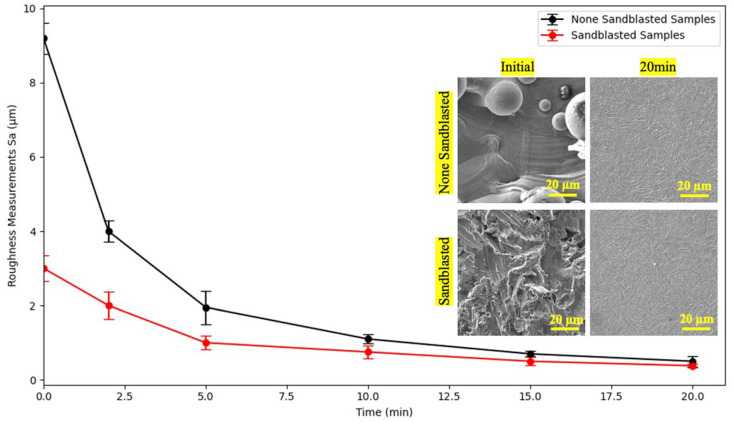
Evolution of surface roughness (Sa) of 3D-Printed Ti-6Al-4V Samples during PEP as a function of polishing time for an as-printed and blasted specimen with close-up scanning electron micrographs from the surfaces at the beginning and at the end of the process, respectively.

The primary challenge in applying the plasma electrolytic polishing process to Ti64 lies in the effective reduction of the surface roughness of 3D-printed samples as seen in Figure 1a, which typically exhibit an initial average roughness (Sa) ranging from 12 to 20 µm [6,7] and in our case of approximately 9.2 μm. Preliminary results from polishing 3D-printed Ti64 samples using the PEP process have demonstrated significant improvements in surface quality (Figure 1b,c and Figure 2) [16]. The most effective outcomes were achieved under conditions that were optimized to include low concentrations of NH_4_F and NH_4_Cl, low operating voltages, and polishing durations of 2, 5, 10, 15, and 20 min. It is noteworthy that a substantial 18-fold reduction in surface roughness was observed under conditions of 300 V for 20 min, yielding a final Sa value of 0.5 μm. For instance, the study post-processing of titanium 3D-printed with radio frequency plasma reports a post-processing technique that requires nearly four hours to reduce the surface roughness only slightly, from 11 µm to 10 µm [30]. In a similar manner, in the post-processing of 3D-printed metal scaffolds, sandblasting and vibratory finishing were employed to modify the surface topography of Ti64 scaffolds. Nevertheless, these conventional techniques remain limited in their ability to achieve substantial roughness reduction, starting from around 20 µm to 5 µm [31].

It is imperative to emphasize that the reduction in surface roughness from approximately 9 µm to as low as 0.4 µm in a single step has been demonstrated exclusively through the plasma electrolytic polishing process. The substantial enhancement in processing efficiency, achieved within a comparatively brief timeframe, underscores the efficiency of PEP in comparison to conventional post-processing methodologies [32,33].

Further investigations are required to comprehensively evaluate the resulting surface quality, encompassing aspects such as microstructural integrity, the chemical composition of the polished layer, and the potential impact on functional performance in demanding applications. However, recent analysis of the leveling mechanism in plasma electrolytic polishing of titanium alloys has been conducted, the results of which indicate that the mechanical structure and surface properties are not deteriorated after PEP [25,34,35]. In contrast, PEP significantly enhances surface properties and the high-cycle fatigue performance of additively manufactured Ti-6Al-4V by generating a surface state less susceptible to crack initiation, thereby improving its mechanical integrity for medical applications [17].

The evolution of surface roughness in Figure 2 during plasma electrolytic polishing of 3D-printed Ti-6Al-4V was systematically investigated under both as-printed and pretreated specimen by blasting for 10 min. Quantitative roughness measurements (Sa) revealed a significant reduction in surface roughness with increasing polishing time, thereby demonstrating the high efficiency of the PEP process. Initially, non-blasted samples exhibited a high roughness of approximately 9.2 µm, while blasted samples started at a lower roughness of around 3 µm due to the mechanical pre-treatment. A rapid decrease in Sa was observed during the initial five minutes of polishing in both conditions, with the non-blasted samples demonstrating a more pronounced decline. Subsequent to a 20-min period, the samples that had not undergone blasting exhibited a final Sa of 0.47 µm, signifying a 20-fold reduction. In contrast, the blasted samples demonstrated a final Sa of 0.38 µm, indicating an 8-fold reduction. These observations are corroborated by scanning electron microscopy (SEM) micrographs, which reveal the removal of partially fused powder particles and surface deformation features.

As indicated by the results of the study, following a 20-min treatment period, both sample types exhibited uniform and smooth textures. Furthermore, cross-sectional analysis revealed that both types of samples experienced a total material removal of approximately 300 µm from the surface (Figure 1c), indicating a substantial yet controlled polishing depth. The presence of broader error bars at early stages, particularly in non-blasted samples, suggests a higher initial surface heterogeneity.

**Figure 3:**
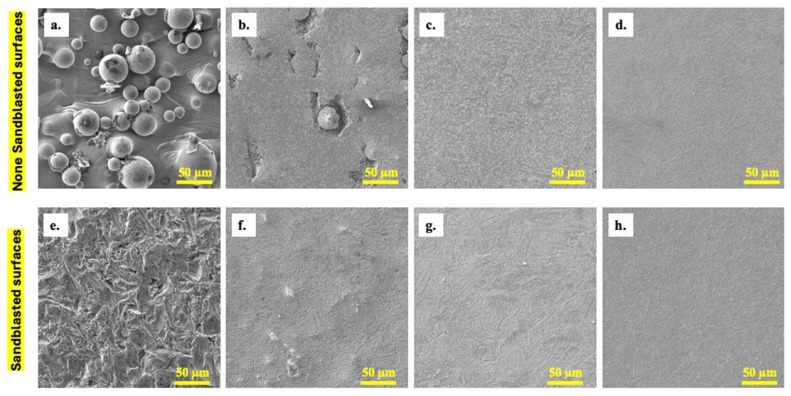
SEM images of 3D-printed samples: (**a**,**e**) initial, (**b**,**f**) after 2 min, (**c**,**g**) after 10 min, and (**d**,**h**) after 20 min of processing without pretreatments and pretreated specimen by blasting in the lower row, respectively.

The scanning electron microscopy (SEM) images in Figure 3 illustrate the progressive surface evolution of 3D-printed Ti-6Al-4V under plasma electrolytic polishing, comparing non-blasted (top row, Figure 3a–d) and blasted (bottom row, Figure 3e–h) conditions. Initially (see Figure 3a,e), non-blasted samples exhibit the typical surface features of additively manufactured materials, dominated by partially fused powder particles and spherical asperities. Conversely, surfaces subjected to blasting exhibit a textured, irregular morphology, a consequence of the mechanical abrasion induced by the blasting process. When exposed to 2 min PEP (see Figure 3b,f), both conditions exhibited early-stage smoothing, with the non-blasted sample still revealing subsurface defects, such as pits and embedded particles, while the blasted sample began to exhibit more uniform dissolution of surface asperities. As the duration of polishing increases to 10 min (see Figure 3c,g), a substantial enhancement in surface homogeneity becomes apparent.

In the case of non-blasted samples, PEP has been shown to effectively remove loosely bonded particles and mitigate microstructural inconsistencies, thereby producing a continuous texture. In samples subjected to blasting, the initial uneven morphology is progressively evened out, suggesting localized plasma activity across the surface. Following extensive polishing of 20 min (Figure 3d,h), both sample types demonstrate a convergence in surface morphology, exhibiting a comparable smoothness characterized by a fine grain-like pattern, devoid of any visible contaminants or residual roughness features. It is noteworthy that the blasted surfaces achieve comparable finishing with slightly fewer intermediate defects. This suggests a potential benefit of pre-treatment in promoting uniform current distribution and stable plasma activity during PEP. The observed trend indicates that PEP demonstrates a high degree of efficacy in the removal of additive manufacturing artifacts and the enhancement of surface quality, irrespective of the pretreatment method. However, the initial topography exerts a substantial influence on the material removal behavior during the initial stage.

## 4. Conclusions

The findings of this study substantiate the efficacy of Plasma Electrolytic Polishing (PEP) in markedly diminishing the surface roughness of 3D-printed Ti-6Al-4V (Ti64) components. As demonstrated by the data, both the as printed and blasted samples exhibited a substantial decrease in surface roughness (Sa) with an increase in polishing time. It is noteworthy that surface finishes as low as 0.38–0.5 μm could be attained, with the duration of treatment extending up to 10–15 min, depending on the initial surface condition. These findings suggest that PEP is a viable surface finishing technique for Ti64 parts requiring low surface finishing, as demonstrated in the results section.

In summary, PEP provides a non-mechanical, efficient, and scalable solution for the polishing of complex geometries in additively manufactured Ti64 parts, rendering it particularly suitable for meeting stringent surface requirements in regulated industries. PEP functions as a solitary post-processing stage, facilitating the attainment of high-end ASME BPE-compliant surfaces and can thus act both as a polishing and finishing process whilst maintaining the homogeneity of dimensional accuracy and the flatness of the samples. This approach eliminates the necessity for costly multi-stage post-processing chains (e.g., grinding + electropolishing + chemical etching), which presently impede the economic viability of 3D-printed Ti64 components. To the best of our knowledge, this study is the first to demonstrate that PEP alone can attain in one step medical/aerospace-grade surface finishes on untreated additively manufactured Ti64, with a significant reduction in time, energy, and cost compared to conventional EP based process.

In consideration of the Ti64 and subsequent developments, the technological implications of the present work highlight the broader potential of PEP as a universal post-processing strategy for advanced manufacturing. Given its electrochemical nature, PEP can in principle be applied to a wide range of conductive alloys, including stainless steels and Co-Cr alloys. These are frequently used in medical, aerospace, and energy applications. Consequently, future investigations may concentrate on adapting electrolyte formulations and process parameters to diverse material classes, thereby facilitating the expansion of PEP to a range of industrial sectors. The provision of a rapid, cost-effective, and single-step finishing method by PEP has the potential to streamline post-processing workflows and accelerate the adoption of metal additive manufacturing across multiple sectors.

## Data Availability

The original contributions presented in this study are included in the article. Further inquiries can be directed to the corresponding author.
